# Pediatric social robotics in ASD: navigating AI blind-spots toward clinical validation

**DOI:** 10.1007/s00431-026-07401-8

**Published:** 2026-09-29

**Authors:** Sahaj Grewal, Raymond Roy, Kiranpreet Sidhu, Kejah Bascon, Feeda Elahresh, Fateh Ahmad, Logendra Naidoo, Amedeo D’Angiulli

**Affiliations:** 1 Dept. of Biomedical Engineering, University of Miami, Miami, FL USA; 2 Neuroscience of Imagination Cognition and Emotion Research (NICER) Lab, Carleton University, Ottawa, ON Canada; 3 Dept. of Neuroscience, Carleton University, Ottawa, ON Canada; 4 Department of Mechanical and Industrial Engineering, University of Toronto, Toronto, ON Canada; 5 Robotics Institute, The Hospital for Sick Children, Toronto, ON Canada; 6 School of Medicine, Wayne State University, Detroit, MI USA; 7 Department of Psychology, International Telematic University Uninettuno, Rome, Italy

**Keywords:** Autism spectrum disorder, Social robotics, Generative AI, Pediatric therapy, Clinical validation, VR–robot hybrids, Wearable sensors

## Abstract

As artificial intelligence becomes increasingly integrated into children’s therapeutic environments, social robots are emerging as one of the most clinically complex AI applications for pediatric autism spectrum disorder (ASD). This mini-review examines three under-validated “blind-spot” domains: robot–wearable sensor integration, immersive VR–robot hybrid systems, and generative AI conversational interfaces. Although these technologies offer promising opportunities for adaptive and personalized intervention, current evidence remains limited by small pilot studies, insufficient longitudinal validation, and unresolved developmental safety concerns. Key risks include pediatric sensor intolerance, cognitive overload during immersive exposure, privacy concerns related to continuous data collection, and unconstrained AI-generated dialogue that may reinforce maladaptive communicative patterns in vulnerable children. We propose a five-module clinical validation roadmap focused on pediatric calibration standards, standardized developmental outcome measures, multisite randomized controlled trials, longitudinal monitoring, and human-supported safety architectures.

*Conclusion*: Adaptive social robotics offers a promising but currently unvalidated frontier in pediatric ASD care. These systems cannot yet be supported as primary therapeutic interventions, as pediatric-specific calibration standards, standardized developmental outcome measures, and adequately powered longitudinal trials remain absent. The proposed validation framework outlines a pathway for ensuring AI-supported interventions augment rather than replace authentic human-centered developmental care.

**Table:** 

**What is Known:** • *Social robots can produce measurable short-term gains in social and communication skills in children with autism spectrum disorder.* • *Supporting evidence derives largely from small, short-duration pilot studies conducted in controlled settings, limiting clinical applicability.*
**What is New:** • *Three under-validated blind-spot domains are identified: robot–wearable sensor integration, VR–robot hybrid systems, and generative AI conversational interfaces.* • *A five-module Clinical Validation Roadmap is proposed to move these domains from pilot demonstration toward pediatric standard of care.*

**What is Known:**

• *Social robots can produce measurable short-term gains in social and communication skills in children with autism spectrum disorder.*

• *Supporting evidence derives largely from small, short-duration pilot studies conducted in controlled settings, limiting clinical applicability.*

**What is New:**

• *Three under-validated blind-spot domains are identified: robot–wearable sensor integration, VR–robot hybrid systems, and generative AI conversational interfaces.*

• *A five-module Clinical Validation Roadmap is proposed to move these domains from pilot demonstration toward pediatric standard of care.*

## Introduction

As artificial intelligence becomes increasingly integrated into daily life, its clinical implications for pediatric populations are acquiring new urgency. Broader pediatric technology literature has already linked structured interaction with educational robotics and digital tools to gains in higher-order cognitive skills, including problem solving, creativity, and critical thinking [1]. However, this only addresses general pediatric and educational populations, leaving a significant gap in available literature on disorder-specific findings. Autism spectrum disorder (ASD), in particular, has become a heightened area of interest because its profound phenotypic, sensory, and communicative heterogeneity has long challenged standardized behavioral interventions. This complexity has encouraged growing clinical and technological interest in autonomous AI-enabled modalities, with three emerging approaches now occupying an important frontier in pediatric ASD therapy: robot and wearable sensor integration, immersive VR and robot hybrid systems, and generative AI conversational interfaces [2].

These interventions represent a shift away from static, pre-programmed therapeutic tools toward adaptive closed-loop systems that can perceive, interpret, and respond to a child’s physiological, behavioral, or cognitive state in real time. While this adaptive capacity may expand therapeutic personalization, it also introduces new clinical risks that are not easily captured by evaluation metrics designed for conventional behavioral tools. These include sensor-induced sensory distress, immersion-related cognitive overload, and unpredictable AI-generated dialogue that may influence children’s communicative development in ways that remain insufficiently understood. As AI applications continue to expand in pediatric settings, the clinical community has a pressing obligation to characterize these risks before widespread deployment.

**Fig. 1:**
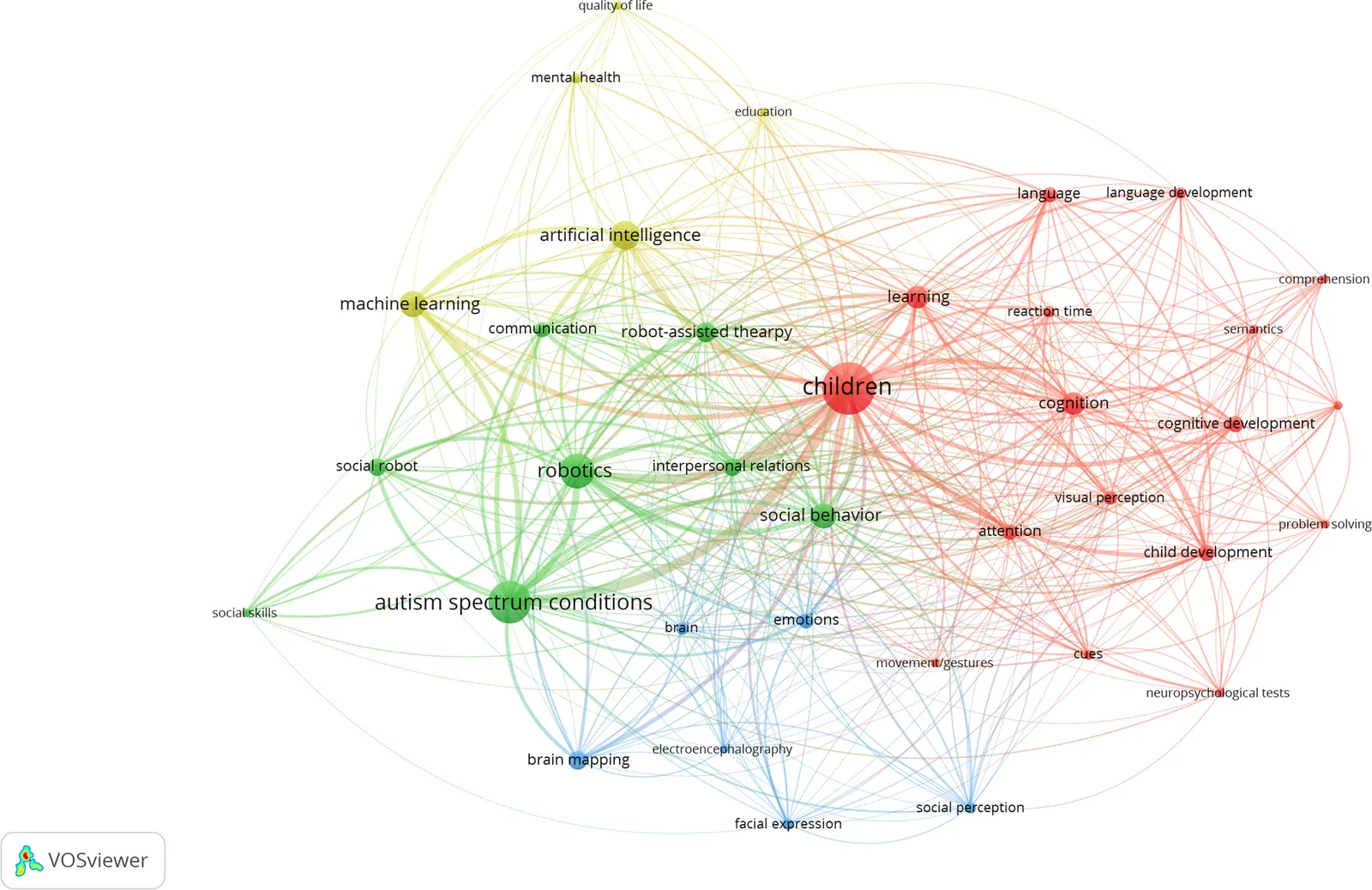
Pediatric and autism-specific robotics network. Bibliometric co-occurrence map generated from 6907 PubMed-indexed records (the pediatric- and autism-specific robotics corpus, which is the pediatric-ASD-specific subset of the 106,907-record dataset characterized in the companion landscape analysis) describing robotic therapeutic tools and AI-assisted interventions for children with autism and related developmental conditions. Nodes represent terms extracted from titles, abstracts, and keywords. Node size reflects term occurrence frequency, while link thickness represents total link strength between co-occurring concepts. Colors denote VOSviewer-generated thematic clusters, with the red cluster emphasizing developmental and cognitive domains (children, cognition, attention, learning), the green cluster linking robotics and therapeutic modalities (robot-assisted therapy, social robot, ASC), the blue cluster focusing on perceptual and affective processes (brain mapping, emotions, social perception), and the yellow cluster highlighting technological and methodological approaches (artificial intelligence, machine learning, communication). The network demonstrates strong conceptual integration between robotics, developmental outcomes, and adaptive therapeutic technologies while also revealing emerging domains where clinical implementation may be advancing more rapidly than mechanistic and long-term empirical understanding. These regions informed the identification of the blind-spot domains discussed throughout this mini-review. The full search strategy and the complete 106,907-record dataset, of which this 6907-record pediatric-ASD corpus is the pediatric- and autism-specific component, are reported in the companion landscape analysis: Mapping the Landscape of Pediatric Social Robotics for Autism: A Quadrant Analysis Across 106,907 Publications

To contextualize these emerging concerns, we generated a bibliometric co-occurrence map from PubMed-indexed literature on pediatric ASD and social robotics using VOSviewer (Fig. 1). This co-occurrence map was constructed from a pediatric- and autism-specific robotics corpus of 6907 PubMed records, the full retrieved set for this domain (English-language records; title, abstract, author-keyword, and MeSH fields; searched 4 December 2025 with no date restriction and deduplicated by DOI and title). These 6907 records constitute the pediatric-ASD-specific component of the larger 106,907-record dataset assembled for our companion landscape analysis, which additionally incorporated two capped reference corpora, namely general therapeutic and rehabilitation robotics and the broader autism literature (50,000 records each). Figure 1 therefore visualizes only the 6907-record pediatric-ASD subset, whereas the figure of 106,907 records denotes the complete three-corpus dataset characterized in that companion analysis. Terms were extracted from titles, abstracts, and keywords, and thematic clusters were derived in VOSviewer using full counting and association-strength normalization. In this review, a “blind spot” refers to a known unknown: an area that is visible within the pediatric robotics literature but remains insufficiently understood, validated, or standardized for clinical implementation. These domains are not absent from the field; rather, they appear as emerging or recognized areas whose empirical depth has not yet matched their conceptual or technological promise. We derived the three blind spots by interpreting the structure of the network, with particular attention to domains that appeared relevant to pediatric ASD care but showed limited mechanistic explanation, long-term validation, safety characterization, or implementation guidance [3]. On this basis, robot and wearable sensor integration, immersive VR and robot hybrid systems, and generative AI conversational interfaces were positioned as clinically relevant blind spots. The accompanying figure summarizes this conceptual placement, and this mini-review focuses on the clinical pathway required to move these three domains from experimental innovation toward responsible integration into standard care.

## Three emerging blind-spots in pediatric social robotics

The technologies and systems discussed across the following three domains are experimental, adjunctive components of therapy rather than independently validated clinical interventions. A systematic review of randomized controlled trials evaluating social robots in autism care illustrates this limitation. While most reviewed trials reported positive outcomes on primary parameters, the broader evidence base remains constrained by a shortage of adequately powered trials, low statistical power, and elevated risks of bias. In practice, these technologies function primarily as engagement or entertainment agents rather than validated standalone therapeutic tools [4]. This systemic lack of evidence underscores the necessity of the roadmap proposed in the “A five-module clinical validation roadmap” section, which outlines a suggested pathway for these blind-spot systems to achieve the clinical validation required to become viable standard of care models in pediatric ASD treatment.

### Robot–wearable sensor integration

Wearable-integrated robots synthesize physiological data, including heart rate, electrodermal activity (EDA), and motion, with behavioral cues such as facial affect and gaze direction to enable adaptive therapeutic responses. Feasibility has been demonstrated: heart-rate features combined with an XGBoost classifier predicted challenging behaviors during robot-assisted ASD sessions with 99% accuracy [5]. These findings were derived from a sample of only five children (all male) however and should be interpreted as a preliminary result that may reflect overfitting rather than established clinical evidence. Longitudinal in-home deployments further confirm that adaptive social robotic systems can sustain engagement across weeks of naturalistic interaction [6].

However, multisensor fusion substantially increases computational load and signal noise, reducing robustness outside controlled environments [7]. Recordings exceeding 8 h are frequently described as burdensome, and device discomfort is common amongst ASD children with sensory sensitivities [8, 9]. Most existing systems treat biosensors as peripheral diagnostic add-ons rather than fully integrated therapeutic components. Clinical translation therefore requires closed-loop adaptation models capable of dynamically adjusting robotic behavior in response to real-time physiological states, alongside pediatric-specific sensor calibration standards.

Equity considerations are equally important. Access to high-cost wearable systems disproportionately benefits well-resourced families and clinical settings, risking the expansion of existing therapeutic disparities across socioeconomic groups. Open-source wearable platforms paired with caregiver-facing dashboards represent a practical pathway toward broader accessibility by enabling teachers, parents, and community caregivers to monitor stress indicators, engagement levels, and behavioral progress in real time across multiple environments, thereby extending the therapeutic reach of these systems beyond the clinic.

### VR–robot hybrid interventions

Hybrid VR and robot systems combine the physical presence of a social robot with immersive virtual environments to support repeated, controlled social skills practice with gradual transfer to real-world settings. Evidence suggests that hybrid formats may provide advantages over either modality independently. Although children often prefer VR during short-term sessions, long-term engagement appears to peak under hybrid conditions [10]. Broader evidence-based reviews of virtual reality interventions in ASD showcase moderate effectiveness across social, cognitive, and behavioral domains [11]. However, controlled pediatric studies have not yet established comparative outcomes, such as narrative engagement or anthropomorphism scores for VR-robot storytelling relative to robot-only approaches. Similarly, the magnitude of anxiety or stress reduction achievable through reinforcement-learning optimization remains unproven and should be regarded as a hypothesis warranting further studies.

Furthermore, significant developmental and clinical constraints remain, primarily due to children with ASD frequently experiencing cognitive overload and fatigue during prolonged VR exposure. This is compounded by there being no established consensus on age-stratified exposure thresholds for this population [11]. Consequently, existing evidence-based reviews of VR interventions recommend that exposure duration be individually monitored according to each child’s tolerance. Sensory sensitivities may further reduce headset tolerance, requiring adaptive systems capable of dynamically adjusting brightness, audio intensity, and break scheduling. A 20-week AR-based imitation program demonstrated observable gains without reaching statistical significance, partly due to a limited sample of six children [10]. Adequately powered randomized controlled trials directly comparing VR-only, robot-only, and hybrid conditions, particularly those evaluating generalization to unstructured human interaction, remain necessary before broader clinical translation can occur.

### Generative AI conversational interfaces

Of the three blind-spots, generative AI conversational interfaces most directly embody the tensions identified in the broader discourse on children growing up with AI. Robots equipped with large language models (LLMs) offer a pathway beyond scripted dialogue trees, enabling adaptive explanations, storytelling, and conversational role-play aligned with therapeutic strategies emphasizing turn-taking and pragmatic language acquisition. A systematic review on chatbot-mediated interactions outside the scope of ASD supports this belief, as researchers found links between structured conversational engagement and measurable benefits towards cognitive control and executive function, suggesting that there could be a plausible mechanistic pathway by which adaptive conversational agents could support the executive-function demands documented in pediatric-ASD populations with further validation [12]. Cumulative evidence supports this claim: robot-based interventions significantly improve communication skills (Hedges’ g = 0.35; 95% CI 0.09–0.61; *p* < 0.01) [13], and a robot interaction partner increased child-initiated verbal utterances toward humans by 26% [14]. Transfer learning applied to child–autism interaction datasets has demonstrated improved dialogue coherence and engagement, with performance metrics outperforming canonical scripted systems across coherence and semantic retention measures [15].

Yet the same adaptive capacity that makes these interfaces therapeutically valuable introduces unique risks in pediatric populations that are both documented and theoretical. A recent scoping review of generative AI applications in ASD assessment and treatment highlighted several limitations across the identified studies. These included small sample sizes, data biases, limited validation, and model hallucinations, noting that most applications remain at an early proof-of-concept stage [16]. We further hypothesize that conversational AI systems could, in principle, amplify a user’s existing beliefs while avoiding dialogue that contradicts their perspective. In theory, this echo chamber risks reinforcing maladaptive interpretations of social cues or inadvertently teaching behaviors incompatible with real-world socialization. Assessing this concern is critical for providing full transparency regarding the technology’s effect on children with ASD, for whom receiving corrective communicative feedback instead of validation is a core therapeutic objective. However, this potential echo chamber dynamic has yet to be empirically demonstrated in pediatric ASD populations and therefore cannot yet be considered an established risk factor. Another concern is that most generative corpora also significantly overrepresent English-language and neurotypical interaction data, limiting applicability across diverse ASD presentations and global populations [17]. Effective deployment therefore demands pediatric-specific training corpora, multi-layer semantic filtering, real-time monitoring, and mandatory human oversight embedded within established frameworks such as applied behavior analysis (ABA) or speech-language pathology.

## A five-module clinical validation roadmap

**Fig. 2:**
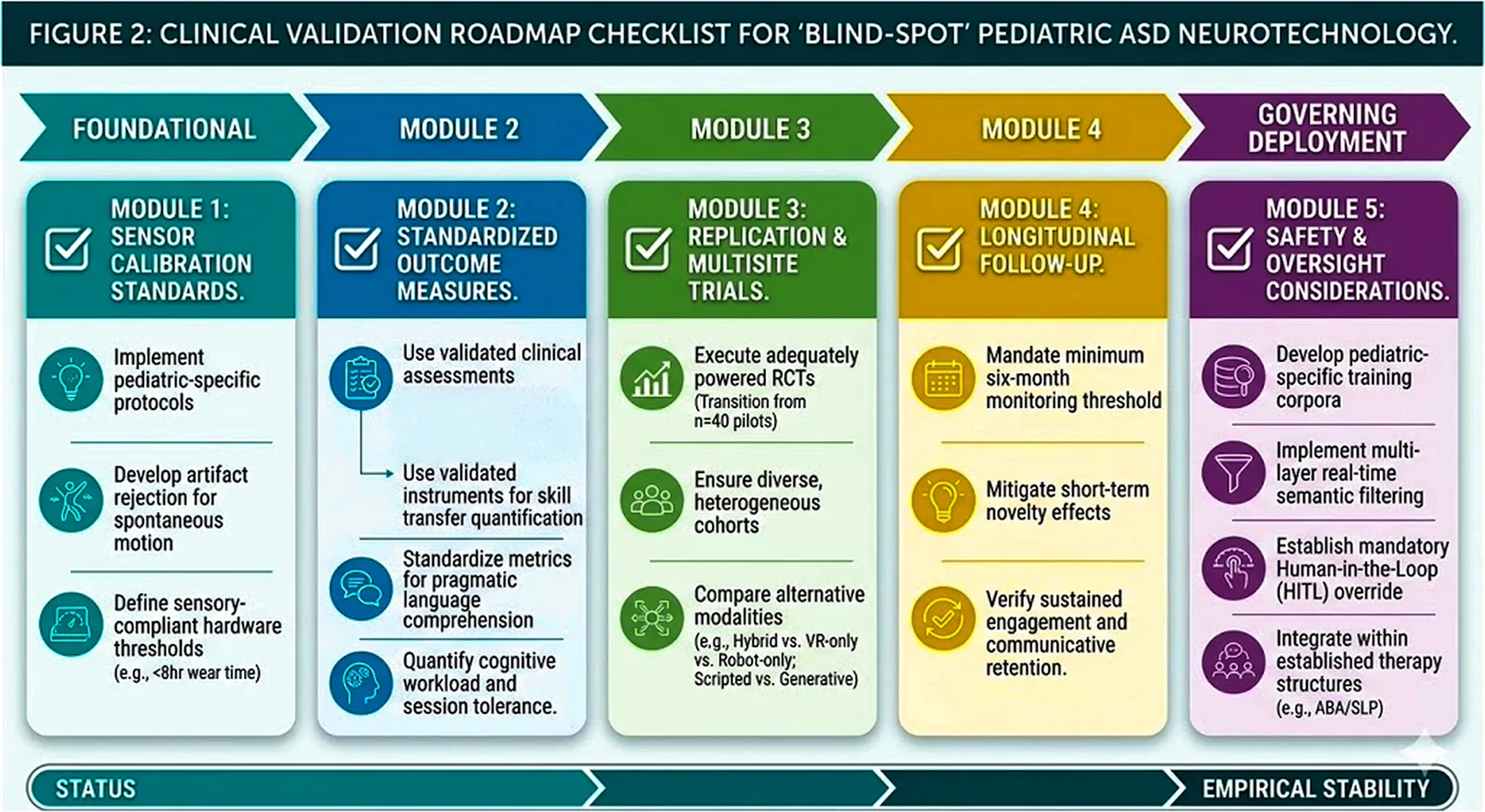
Clinical validation roadmap for blind-spot pediatric social robotics*. *A structured sequential framework is proposed to guide the transition of emerging adaptive interventions, specifically robot and wearable sensor integration, VR and robot hybrid systems, and generative AI conversational interfaces, from experimental prototypes toward scalable standards of care. The roadmap begins with a foundational phase that addresses hardware variability and evaluation inconsistencies through pediatric-specific sensor calibration standards designed to reduce artifact noise from spontaneous movement, alongside standardized clinical assessments capable of quantifying cognitive workload, sensory burden, and real-world skill generalization. The second phase emphasizes empirical validation through adequately powered multisite randomized controlled trials involving heterogeneous pediatric cohorts, combined with longitudinal monitoring periods of at least 6 months to distinguish durable communicative retention from short-term novelty effects. The final deployment phase establishes the safety and governance architecture necessary for partially autonomous therapeutic systems, including pediatric-specific training corpora, multi-layered real-time semantic filtering, and mandatory human-in-the-loop override mechanisms. Integration within established therapeutic structures, such asapplied behavior analysis (ABA) and speech-language pathology (SLP), further supports clinical oversight and developmental appropriateness. Collectively, this framework provides a translational pathway for addressing the current empirical and mechanistic gaps limiting the broader clinical adoption of adaptive social robotics tools in pediatric ASD care

The three blind-spot modalities share a structural problem in that current evidence rests on short-term pilot studies with small, homogeneous samples that capture transient engagement while failing to characterize safety profiles, developmental generalization, or long-term therapeutic durability. Translating these technologies into scalable standard-of-care models requires addressing these gaps in a coordinated, sequential manner. Figure 2 and Table 1 present the proposed five-module clinical validation roadmap, which reframes existing empirical gaps as actionable clinical priorities.

1. Pediatric Sensor Calibration Standards.

Multisensor fusion protocols must be validated against pediatric-specific motor and behavioral profiles, with artifact rejection algorithms designed to account for the spontaneous movement patterns and sensory variability commonly associated with ASD [7, 18]. VR calibration standards should also dynamically adjust brightness, audio intensity, and break pacing according to developmental stage, with clearly defined age-stratified exposure limits to reduce fatigue and cognitive overload.

Module 2: Standardized outcome measures

Evaluation frameworks must move beyond case-specific laboratory metrics, such as raw gaze duration or algorithmic fluency, toward standardized clinical instruments capable of assessing real-world skill generalization and functional developmental outcomes, including measures such as the ADOS-2 and VABS-3 [19]. For generative AI interfaces specifically, outcome measures should assess pragmatic comprehension, semantic retention, conversational reciprocity, and naturalistic communicative development rather than simple verbal initiation counts alone.

Module 3: Multisite randomized controlled trials

Current wearable and AR studies, many of which involve approximately 40 participants or fewer, remain insufficient to support broad clinical claims. Adequately powered multisite randomized controlled trials are therefore required to account for the substantial phenotypic, developmental, and socioeconomic heterogeneity characteristic of ASD populations [2]. Such studies are essential for establishing empirical stability, reproducibility, and broader clinical validity.

Module 4: Longitudinal monitoring (≥6 months)

Short-term pilot studies cannot reliably distinguish durable skill acquisition from novelty-driven engagement effects. The novelty effect, in which initial enthusiasm toward a new robotic system temporarily inflates engagement metrics despite limited long-term learning, remains a well-recognized confound within pediatric robotics research [6]. Empirically, novelty-driven engagement in pediatric human–robot interaction typically attenuates within the first weeks to months of repeated exposure [6], meaning that observation windows shorter than this interval risk conflating transient interest with therapeutic effect. A 6-month minimum extends well beyond this decay window and approximates one full academic term, allowing assessment to span the developmental, educational, and seasonal transitions during which behavioral generalization is most likely to be tested. Where feasible, follow-up should also be developmentally indexed rather than purely calendar-fixed, for example, anchored to school-term boundaries or to pre- and post-transition points around key developmental milestones, with recurring socio-behavioral assessments coordinated through the child’s primary care team to evaluate therapeutic durability across developmental stages.

Module 5: Human-supported safety architecture

Generative AI and VR-based systems must incorporate multi-layer semantic filtering, pediatric-specific training corpora, and real-time clinician override capabilities. Integration within established therapeutic frameworks, including applied behavior analysis (ABA) and speech-language pathology (SLP), helps maintain developmental appropriateness, clinical oversight, regulatory compliance, and long-term clinician confidence [17]. These safeguards must also align with existing regulatory frameworks for clinical software. Adaptive pediatric robotics systems generally fall within the scope of Software as a Medical Device (SaMD) classification under the U.S. Food and Drug Administration, and within Medical Device Regulation (MDR) requirements for CE marking in the European Union, while their embedded software components are expected to conform to recognized lifecycle and risk management standards such as IEC 62304 and ISO 14971. Pediatric deployment imposes additional constraints: safety and efficacy data generated in adult populations cannot be extrapolated to children without dedicated pediatric validation, particularly given the developmental sensitivity of sensory tolerance, cognitive load, and communicative outcomes emphasized throughout this review. Human-in-the-loop architectures should therefore be designed not only as clinical safeguards but as auditable mechanisms that satisfy post-market surveillance and adverse event reporting obligations under these frameworks. Together, these safeguards establish the governance architecture necessary for the responsible deployment of adaptive pediatric social robotics systems.

**Table 1:** Five-module clinical validation roadmap for blind-spot pediatric social robotics in ASD

Module	Focus area	Core requirement	Target modality
1	Pediatric sensor calibration standards	Artifact rejection algorithms validated against ASD-specific motor profiles; age-stratified VR sensory parameters (brightness, audio, break pacing)	*Robot–wearable; VR–robot*
2	Standardized outcome measures	Validated clinical instruments (ADOS-2, VABS-3, pragmatic language assessments); real-world skill generalization, not laboratory proxies	*All three modalities*
3	Multisite randomized controlled trials	Adequately powered, phenotypically and socioeconomically diverse cohorts; active comparators (VR-only vs. robot-only vs. hybrid conditions)	*All three modalities*
4	Longitudinal follow-up (≥ 6 months)	Biannual physician-led socio-behavioral assessments; distinguish habituation and novelty effects from durable skill acquisition; monitor developmental stage transitions	*All three modalities*
5	Human-supported safety architecture	Pediatric-specific multilingual training corpora; real-time multi-layer semantic filtering; clinician override capability; ABA/SLP integration; alignment with SaMD/MDR and IEC 62304 lifecycle standards	*Generative AI; VR–robot*

*ABA*, applied behavior analysis;SLP, speech-language pathology; ADOS-2, Autism Diagnostic Observation Schedule, 2nd ed.;* VABS-3*, Vineland Adaptive Behavior Scales, 3rd ed.;* HITL*, human-in-the-loop

## Ethical and developmental considerations

The integration of AI-enabled social robots into pediatric ASD therapy raises substantive ethical concerns that align directly with the broader “Growing Up with AI” discourse. Firstly, concerns regarding data privacy must still be addressed, as wearable biosensors continuously collect sensitive physiological data from children; often without adequate parental or child understanding of its use, storage, or transmission which raises concerns analogous to those surrounding consumer-facing AI data collection [17]. Secondly, algorithmic bias is still a factor, with most models trained on demographically narrow or English-dominant corpora, which pose the risk of widening therapeutic inequity across racial, linguistic, and socioeconomic lines. Finally, and perhaps most critically for child development, generative AI interfaces risk simulating meaningful social interaction without fostering genuine communicative growth or authentic human connection. Research consistently demonstrates that interacting with AI assistants does not generate real emotional bonds or develop the reciprocal social skills that are central therapeutic targets in ASD [13, 14].

These systems must therefore be positioned unambiguously as therapeutic adjuncts that augment, rather than substitute, human interaction. Pediatricians, child neurologists, and developmental specialists are uniquely placed to provide the oversight necessary to ensure that AI-enabled tools serve children’s authentic developmental goals and maintain appropriate boundaries between technological frameworks and genuine social learning while protecting vulnerable pediatric populations from the unintended consequences of premature autonomous AI deployment.

## Conclusion

Several methodological boundaries of this review warrant acknowledgment. The framework presented is derived exclusively from pediatric ASD populations; therefore, the calibration thresholds, dosing parameters, and safety architectures proposed here cannot be generalized to adult neurorehabilitation or other neurodevelopmental conditions without separate empirical validation. The roadmap also remains a theoretical construct, structurally derived from fragmented pilot data, and will require prospective testing across controlled multisite environments before it can serve as a trusted clinical blueprint.

The blind-spot technologies examined here, namely wearable sensor fusion, VR and robot hybrid systems, and generative AI conversational interfaces, represent the most dynamic and potentially transformative frontier in pediatric ASD therapy. Their adaptive architectures offer unprecedented personalization for heterogeneous populations that standardized behavioral tools have not fully served. However, premature clinical deployment before resolving sensor tolerability, cognitive overload, and unconstrained AI outputs risks causing acute harm, including sensory distress, device abandonment, and behavioral regression in vulnerable pediatric populations. This concern is especially important because generative AI systems can produce increasingly sophisticated representations and language-like outputs without necessarily possessing biological understanding, affective grounding, or self-regulating awareness [20, 21].

The proposed five-module clinical validation roadmap provides a sequential and actionable pathway for converting recognized “known unknowns” into evidence-based, scalable therapeutic tools. As AI increasingly shapes children’s developmental environments across clinical, educational, and home settings, the pediatric medical community must insist on rigorous empirical validation, robust safety architectures, and sustained human oversight to ensure that technological innovation supports, rather than compromises, the authentic developmental trajectory of every child on the autism spectrum. In this sense, pediatric social robotics is not a ship that should be launched at full speed simply because its engines are powerful. It is a vessel entering fog dense waters, where every sensor, algorithm, and therapeutic decision must be carefully calibrated before it can safely carry children toward meaningful growth.

## Data Availability

The full dataset used for network construction can be found here under the files who's labels begin with "F2…": https://doi.org/10.5281/zenodo.17821797.
